# 

**Published:** 1957-10

**Authors:** 


### Contents

| | | Page |
|---|---|---|
| CLINICAL PATHOLOGY AND GENERAL PRACTICE: | *Dr. H. J. Gibson* | 109 |
| TOWARDS BETTER HEALTH AT WORK | *Dr. T. G. Faulkner Hudson* | 117 |
| CHILD ART . . . . . | *Mr. D. E. Milner* | 125 |
| ACUTE NECROSIS OF THE LIVER IN PREGNANCY | *Clinical Pathological Conference* | 129 |
| MEMBERS OF THE BRISTOL MEDICO-CHIRURGICAL SOCIETY . | | 133 |
| SUBSCRIBERS TO THE "JOURNAL" . . . . . | | 140 |
| NEWS FROM SOCIETIES . . . . . . | | 141 |
| APPOINTMENTS . . . . . . . . | | 145 |

